# Assessment of technology-based options for climate neutrality in Austrian manufacturing industry

**DOI:** 10.1016/j.heliyon.2024.e25382

**Published:** 2024-02-01

**Authors:** P. Nagovnak, C. Schützenhofer, M. Rahnama Mobarakeh, R. Cvetkovska, S. Stortecky, A. Hainoun, V. Alton, T. Kienberger

**Affiliations:** a Chair of Energy Network Technology, Montanuniversitaet Leoben, Franz-Josef Straße 18, A-8700, Leoben, Austria; b Austrian Institute of Technology, Giefinggasse 4, A-1210, Vienna, Austria

**Keywords:** Industrial climate neutrality, Integrated energy systems, Clean manufacturing, Pathways for industry transition, Scenario modelling

## Abstract

The goals set forth by the European Green Deal require extensive preparation and coordination of all stakeholders. As a valuable tool, energy scenarios can generate the necessary information for stakeholders to envision the right steps in preparing this transition. The manufacturing industries represent an especially important sector to investigate. They are responsible for both high energy consumption and GHG emission figures on the one hand side and provide great economic value for member countries on the other. We aim to provide a close investigation of all thirteen industrial subsectors that can be used as a solid information basis both for stakeholders within the manufacturing industries and policymakers. Our approach includes *all* industrial production processes. We achieve this by considering both transformation processes, such as blast furnaces or industrial power plants, and final energy-application. In addition, both scope 1 and 2 emissions of manufacturing industry are assessed in an effort to transparently indicate the interdependencies of industrial decarbonisation efforts with the overall energy system. We propose the integration of a novel stakeholder-based scenario, that puts special emphasis on first-hand information on mid to long-term planning of key industrial representatives, thereby going beyond existing scenario narratives (e.g., scenarios according to the European Monitoring Mechanism). Thus, a balanced deep decarbonisation scenario using best-available technologies can be compared with existing industry plans. To address these points, we have chosen Austria as a case study. Results indicate that industry stakeholders are in general agreement on their subsector-specific technology deployment and already envision investments towards a low-carbon pathway for their respective subsectors. While today's manufacturing industries rely at large on a great diversity of (mostly fossil) energy carrier supply, deeply decarbonised manufacturing industries of the future may be based on the following main energy carriers; electricity, CO_2_-neutral gases, and biomass. To mitigate emissions from geogenic sources, carbon capture technologies are needed. On the other hand, the synthesis of olefins in the chemical industry may provide a sink for CO_2_ assuming long-term use after production. In addition to the option of using it across subsectors, captured CO_2_ will have to be stored or sold to other economies. Comparison of the developed scenarios allows the identification of no-regret measures to enable climate neutrality by 2050 that should be deployed as soon as possible by push and pull incentives. The model results of the two transition scenarios show the need for technology promotion as well as infrastructure development needs and allow the identification of possible corridors, focal points, and fuel shifts – on the subsector level as well as in energy policy. Among others, the modelled magnitude of renewable energy consumption shows the need for swift expansion of existing national renewable energy potentials and energy infrastructure, especially for energy intensive industry regions. In light of the current energy consumption in other economic sectors (most notably in buildings or transport) and limited renewable potentials, large import shares of national gross domestic energy consumption are likely for Austria in the future.

## Highlights

• Subsector-resolved scenario analysis including bottom-up modelling of energy-intensive manufacturing processes in Austria.
• Industries’ perspective is compared to a backcasting scenario using breakthrough technologies to achieve climate neutrality.
• When considering scope 2 emissions, industries are close to net zero through three dominating technological levers.
• Results identify no-regret strategies for breakthrough technologies’ integration that should be deployed as soon as possible.

## Abbreviations

AWF: Alternative waste fuels
BF/BOF: Blast furnace/basic oxygen furnace
BAT: Best available technologies
BAU: Business as usual
BEP: Break-even point
BTT: Breakthrough technologies
Bio-CH_4_: Biogenic methane
CH_4_-DR-EAF: Methane-based direct reduction and electric arc furnace
CHP: Combined heat and power
DR: Direct reduction
EAF: Electric arc furnace
ENTSO-E: European Network of Transmission System Operators for Electricity
EU: European Union
GDP: Gross domestic product
GHG: Greenhouse gas
H-DR-EAF: Hydrogen-based direct reduction and electric arc furnace
HFC: Hydrofluorocarbons
IT: Information technology
PFC: Perfluorocarbons
POI: Pathway of industry
RES: Renewable energy sources
Syn-CH_4_: Synthetically produced methane
UBA: *german:* Umweltbundesamt – the Austrian environmental agency
UN: United Nations
ZEM: Zero emission

## 1 Introduction

With the Green Deal, the European Union and its member states have set the goal of achieving climate neutrality by 2050 [1]. This requires extensive preparation and coordination of all stakeholders due to the high level of complexity arising from the multitude of involved levels in the economic system. Chiodi et al. [2] review four case studies on the national and supranational level where energy systems models’ scenario output was able to directly help in overcoming barriers in acceptance, fostering understanding of key areas of action and subsequently leading into long-term energy and emission strategy development and impact appraisal thereof. In the European Union, the European Commission names the EU reference scenario one of its “key analysis tools in the areas of energy, transport and climate action” [3]. Together with the array of progress reports on national energy and climate plans filed in line with the “Regulation on the Governance of the Energy Union” [4] and additional national scenario analyses the member states can bring into the policy delivery process, the scenario has also been used as a baseline for the policy initiatives in the “European Green Deal” package. These examples show that the development of energy scenarios is a powerful and proven tool for identifying potentials, envisioning transformation pathways and appraise the success of already adopted measures that needs to be further expanded and continuously adapted to the evolving realities of technological and economic development.

In 2021, manufacturing industries including construction were responsible for 23 % of energy and process-related European greenhouse gas (GHG) emissions, due to the sector's large dependency on fossil energy for energy-intensive production processes [5]. The total impact of the sector on GHG emissions of the EU are even greater due to upstream electricity and heat generation. On the other hand, the manufacturing industries is an important part of the Union's economy, directly accounting for approximately 15 % of value added and employing 16 % of its workforce [6,7]. Therefore, finding sustainable transition pathways for the manufacturing industry is a key for long-term European prosperity in light of the climate crisis [8].

Following the above-described importance of energy systems modelling for policymaking, energy consumption and accompanying GHG emission scenarios of manufacturing industries can help to envision successful pathways to climate neutrality and identify important fields of action. The comparison of meaningful scenario results indicates the bandwidth of possible pathways towards industrial climate neutrality and allows for the identification of technology and non-technology related no-regret measures on this journey. These measures are necessary to transform the manufacturing industry under the assumption that production activities are maintained and not moved abroad.

Due to their important role in understanding the complex challenges of industrial climate neutrality and their potential for providing visionary guidelines, a great diversity of scenario publications on industrial development exists.

Several academic publications focus only on industrial final energy consumption in total energy system analyses. Exemplary for this group, the works of Saddler et al. [9] and Gaur et al. [10] provide energy scenarios for deep decarbonisation of the Australian and Irish energy system. While taking into account industrial final energy consumption and resulting energy-related emissions, energy consumption of industrial energy transformation units and process-related emissions, e.g., from primary steel or cement production, are not considered. Wiese et al. [11] summarise this problem in their meta-analysis of German energy scenarios, noting significant differences in the observed studies “regarding […] process emissions in the industry sector”. This is the case especially for economies with a considerable share of primary production in the energy intensive subsectors.

Other publications, often also in grey literature, remain specific to one industrial subsector or product. This includes roadmaps and technology reports, e.g., by industrial interest groups. For example, in the chemical and petrochemical industry, DECHEMA [12] for Germany and Windsperger et al. [13] for Austria have provided stakeholder roadmaps for the sector's future development. Griffin et al. [14] have contributed scientific investigations for the chemicals sector in the United Kingdom. For iron and steel, the EU Commission has outlined a “future for steel in Europe”, highlighting technology options necessary for the transformation towards climate neutrality [15]. Scientifically, great attention has been given to the question of decarbonising primary steel production through the use of hydrogen direct reduction in combination with electric arc furnaces (H-DR-EAF) as reviewed by Wang et al. [16]. While providing the necessary industrial subsectoral level of detail, these studies lack the possibility to be connected to a greater picture regarding the challenges of attaining industrial climate neutrality because their methodologies and applied balance borders are limited to the specific subsector under investigation and therefore differ greatly.

Some studies on the transformation towards climate neutrality investigate and discuss the role of a single specialised or very limited number of technology pathways, e.g. electrification [17,18], the use of biomass [19,20] or hydrogen [21]. However, due to the much wider range of process technologies actually available to manufacturing industries, these studies cannot depict a holistic transition pathway for entire subsectors or the overall sector.

In comparison to the aforementioned publications, studies focused on several or all subsectors of manufacturing industry considering a large array of technology pathways can present a broader, more holistic overview of transformational options in manufacturing industries. In many cases, due to their large lever towards climate neutrality, the energy-intensive industries iron and steel, chemicals and non-metallic minerals are investigated more deeply by also taking into account process-related energy consumption and emissions. For example, Sánchez Diéguez et al. [22] provide four distinct technology-driven scenarios for the total of Dutch manufacturing industries. Similarly, Fleiter et al. [23] and Schneider et al. [24] use bottom-up modelling tools to calculate transition scenarios for the German industry, taking into account stakeholder information on process peculiarities in the modelling phase based on preliminary results. These and subsequent analyses for Germany are generally embedded in an analysis of the overall energy system making use of a combination of modelling tools [25,26]. This approach merits the big advantage of providing a one-stop-shop for policymakers and a bird's eye view of the energy system under the given scenario narratives – something that is extremely useful in times of uncertainty and demand for quick action. These studies can further be aided by stand-alone publications that are able to underline the lever of technologies and energy carriers of one specific economic sector, in this case the manufacturing industries. This enables a broader stakeholder community – besides policymakers also industrial decision makers and technology officers – to investigate the impact of technology choices in their specific subsectors also when they are not part of the energy intensive industries.

Regarding the formulation of scenario narratives, to the best of our knowledge, industrial stakeholder integration into scenario development has never gone beyond the stage of consultations based on preliminary results of already determined scenario narratives. In above-mentioned group of publications, scenario narratives are focused on emphases on the deployment or sourcing of specific energy carriers (e.g., import/export, electrification, e-fuels), target states (e.g., climate neutrality or GHG reduction by target year) or a combination of these target narratives.

Stakeholder interaction has been recognised as an established and essential tool in all these modelling projects to ensure applicability, understanding and acceptance. However, stakeholder interaction, especially with regards to the manufacturing industries, has not been extended to find reflection in the form of a scenario focus. Under the impression of increasing national and international legislature with regards to GHG mitigation and energy efficiency, many industrial stakeholders already have transition plans on their tables. Their application into scenario modelling and subsequent unioning into subsectorspecific or subsector-overarching scenario results can reduce the risk of developing visions of a climate-neutral future that significantly differ from the industrial realities. In addition, comparison of the resulting pathway of industrial stakeholders with the desired climate neutrality scenarios can enable identification of manufacturing industries’ needs to ensure successful transition.

Because of the apparent large lever of action in the industrial sector, reflected by the multitude of studies and approaches mentioned above, it is important to gain a maximum of subsectoral detail while at the same time preserving the possibility for a broader systemic analysis. Therefore, we deduce that the development of innovative and need-orientated industry pathways with a maximum of information content for decision makers must include the following.

• A subsector-resolved analysis of the whole manufacturing industries sector. Homogenous subsectors – e.g., the iron and steel or the chemical and petrochemical industries – must be investigated on the level of their most dominant industrial production processes considering both transformation processes and final energy-application.
• An indication of resulting energy-related and process-related GHG emissions per subsector, both directly within industrial production and the upstream energy supply sector.
• A methodological integration of industrial stakeholders by means of an “industry scenario” which reflects first-hand industrial development expectations. The aim is to guarantee applicability of results in all subsectors both for industrial and political decisionmakers and prepare the basis for the subsequent implementation of developed pathways towards climate neutrality. By considering first-hand information on already planned transformations, this approach significantly differs from a business-as-usual scenario or “with existing or additional measures” - scenario approaches which have already been often used in literature to assess the changes brought about by the planned policy measures.

To address these points, we have chosen Austria as a case study. With industries’ contribution to national GDP of approximately 25 % well above the European average and high shares of energy intensive primary industries, Austria is a prime example for a highly industrialised economy. In Austria, total manufacturing industry 2019 accounted for approximately 27 % (133 TWh/a) of national gross domestic energy consumption when adding transformation input to combined heat and power plants (CHPs), blast furnaces (BF), coke ovens, and for chemical production to final energy consumption [27]. At the same time, the sector directly contributed approximately 34 % (27 Mt CO_2_e) of national GHG emissions [28].

In section 2, we present and explain in detail the applied methodology of devising energy consumption scenarios and calculating associated GHG emissions for the case study. Subsequently, in section 3, the results of our scenarios for the total of Austrian manufacturing industries are shown and comparatively discussed before we discuss current limitations and necessary future work in the field of manufacturing industries and finally conclude this work in section 4.

## 2 Methodology

For the definition of our subject of investigation we have used the 13 subsectors of manufacturing industries as classified by the United Nations and shown in Table 1 [29].

**Table 1:** Applied division of manufacturing industries into subsectors [29].

Subsector
Iron and steel
Chemical and petrochemical
Non-ferrous metals
Non-metallic minerals
Transport equipment
Machinery
Mining and quarrying
Food and tobacco
Paper, pulp and print
Wood and wood products
Textile and leather
Construction
Industries not elsewhere specified

In this section, the general scenario development methodology is discussed, and important aspects are highlighted specifically. In section 2.1, the chosen balance border is presented to account for the overall energy demand in connection with manufacturing activity. The calculation of energy and GHG emission scenarios follows a two-step process. First, in section 2.2, the chosen scenario storylines are discussed which set the basis for the calculation of the technology-resolved energy consumption results. The resulting energy demands by subsector represent only demands – without consideration of limiting factors such as infrastructure or energy resource availability which are considered in the discussion of results in section 3. Due to the close interlinkage of the manufacturing industries’ energy sector with the overall energy system, it is necessary to apply assumptions on the GHG intensity of the electricity and gas grids in a subsequent step. The applied methodology and results for this step are presented in section 2.3.

Further details are available in the report of the corresponding project NEFI – New Energy for Industry [30], in the course of which this work was carried out.

### 2.1 Application of an integrative balance border

**Fig. 1:**
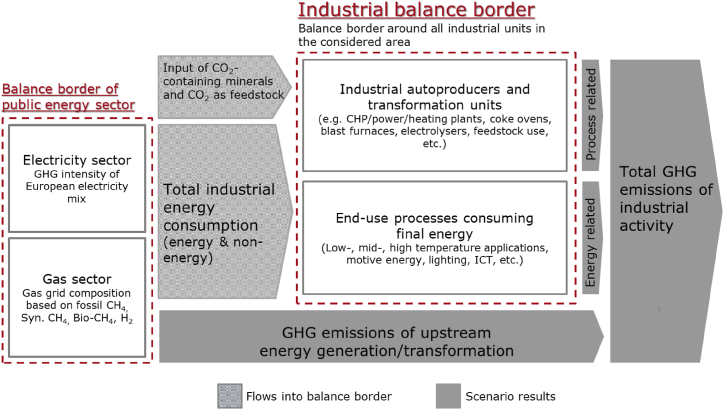
Considered balance border of manufacturing industries and relevant upstream processes.

The investigations within this work are based on a balance border around the industrial sub-sectors (cf. Fig. 1). This allows the examination of both total energy consumption and emissions in each of the industrial subsectors *and* the total of manufacturing industries per reference year. To be able to devise subsector-specific pathways towards climate neutrality, a thorough understanding of their process route related challenges and opportunities is essential. This can only be achieved when the investigated balance border is clearly defined and includes the whole manufacturing activity including both final energy applications and transformation units. Fig. 1 depicts the relevant processes for industrial energy consumption and GHG emissions. Herein, for the first time, industry scenarios are prepared following the proposed improvements to international standards of energy balances proposed by the authors in Ref. [31].

Total industrial energy consumption is, as mentioned, determined in situ by two general consumer categories located inside the balance border surrounding all industrially owned final energy use and transformation units. On one side, energy is used by end-use devices consuming final energy, such as boilers, furnace, engines, or lighting devices. For their application, we have defined five energy application categories – low, medium, and high temperature thermal demand, motive power demand and energy demand for lighting and information technology. On the other side, industries utilise energy for their energy transformation units, e.g., CHP or power plants, blast furnaces, coke ovens, or electrolysers, and as a non-energy use feedstock, e.g., methane or hydrogen for the production of chemicals [30]. Due to the large synergies and future deepening integration into industrial processes, these energy conversion facilities must be included in an industrial energy system model. However, many of these units (e.g., electrolysers) may in the future be operated either inside the presented industrial balance border or inside the energy sector. Therefore, in the results section, we present this energy consumption in the case of electrolysis for hydrogen production in shaded bars as it is directly affecting the magnitude of industrial decarbonisation efforts.

Investigated GHG emissions comprise energy-related and process-related emissions. Energy-related emissions stem from the combustion of fossil energy carriers. Process-related emissions are caused by industrial energy transformation processes (e.g. blast furnace or chemical production (especially CH_4_ and N_2_O) in line with official methodology of the Austrian national GHG emission inventory report [28]) or by carbonaceous minerals in the production processes (e.g. CaCO_3_ for cement production). Additional emission sources (e.g., from product use, for example hydrofluorocarbons (HFC), or minor emissions of perfluorocarbons (PFC), SF_6_, or NF_3_) are not investigated in this study. Besides alternative production technologies and energy carriers, also carbon capture technologies can reduce the GHG intensity of a subsector (e.g., non-metallic minerals). In the chemical industry, CO_2_ may be used as an alternative carbon source in the future in which case we count this CO_2_ as negative emissions in the chemical subsector. Special care must be taken when calculating the aggregate CO_2_ emissions of manufacturing industries. In this case, captured CO_2_ from a subsector employing carbon capture but not directly using this CO_2_ within its own balance border and instead passing it on within the balance border of manufacturing industries must not be double counted.

As pointed out in the NEFI project report [30], upstream production of energy carriers in the public energy sector, for example through the combustion of gas for electricity generation, may add to the GHG intensity of industrial production. We have followed internationally established practice of companies reporting these *scope 2* emissions under energy audit regulations [32]. Therefore, while not directly inside the industrial balance border, these emissions are included in the investigation of industry transformation to avoid moving the challenge of decarbonisation from one sector to another.

Based on the above-described balance border, each subsector is analysed for its current energy consumption, the supplied useful energy categories and transformation processes as well as related GHG emissions. While all subsectors are investigated separately, the homogenous and energy-intensive subsectors iron and steel, chemical and petrochemical industry, the non-metallic minerals and paper, pulp and print are investigated in addition on the level of deployed production processes (e.g. blast furnaces, steam reformers, CHP plants, etc.) Table 2 provides an overview of the considered processes in these sectors. The change in modelled production activity, which is an essential factor for these investigations and has therefore been included in the stakeholder consultation process, are given in Figure A 6 and Table A 1 in the appendix, respectively. Circular economy measures, on the other hand, are not considered in the herein presented investigation.

**Table 2:** Overview of applied investigation level by industrial subsectors.

Industry subsector	Bottom-up modelled manufacturing units	Top-down modelled energy application
Iron and steel	Primary and secondary steelmaking including downstream processing	
Chemical and petrochemical	Ammonia Nitric acid, urea, fertiliser Methanol Olefins	
Non-metallic minerals	Cement production Magnesia production	Clay, glass, lime, ceramics, and auxiliary energy application
Paper, pulp and print	Paper/pulp production, including CHP deployment at integrated plant sites	
Remaining subsectors (non-energy intensive, heterogenous)		Fully modelled top-down based on energy application categories

The employed analysis is based on statistical information available from the Austrian statistics authority Statistik Austria and subsector-specific reports, e.g., from subsector interest groups. In addition, scientific literature on best available (BAT) and breakthrough technologies (BTT) is consulted which has been investigated by the authors in previous works [33,34]. Investigation of the status quo is further strengthened with extensive desk research and expert interviews regarding technological options for GHG mitigation and climate neutrality. The compiled subsector briefings can be found in the project report [30]. The obtained picture of industry subsectors serves as the basis for the subsequent scenario development.

In the iron and steel subsector, two different steelmaking technologies are currently used in Austria: primary steelmaking using the blast furnace/basic oxygen furnace route (BF/BOF) and secondary steelmaking using EAFs. Between 2017 and 2019, the Austrian steel industry produced between 6.9 Mt (2018) and 8.1 Mt (2017) of crude steel per year, of which 90 % was manufactured via the BF/BOF and 10 % via the EAF route [35]. For scenario modelling in this subsector, the total steel production as well as the share of secondary steelmaking by way of EAF through to 2050 is kept constant throughout the modelled timeline to the value of 2017 production in accordance with the communicated plans of the only Austrian company in primary steel production (cf. subsector details in the appendix).

The production of ammonia, urea, fertiliser, nitric acid, methanol and olefins is modelled bottom-up to account for the non-energy consumption of approximately 44 % of the subsector's total energy consumption with activity rates taken from Windsperger et al. [13]. From its total energy carrier consumption of 30 TWh in 2019 over 95 % is currently fossil, mostly naphtha and natural gas. The subsector's production output is set to increase annually by 1.3 % on average in the model. Concordant standard statistical classification, we do not include refineries in the chemical and petrochemical industry as it is accounted for in the energy industries [29].

The Austrian non-metallic minerals subsector represents the second-highest subsector emissions in Austria and can be divided into several areas of production. The production of cement and magnesia specifically accounts for more than 70 % of the total subsector's emissions [28]. Due to their high share of geogenic emissions from mineral resources, these manufacturing units are modelled bottom-up while the remaining energy areas of production are investigated based on their areas of energy application. In general, under consideration of stakeholder feedback on expected activity, production output was kept constant at the levels of the recent past (2017–2019) as published in the Austrian national GHG inventory report [28].

With a total energy demand of over 22 TWh/a, paper, pulp and print is the second most energy-intensive industrial subsector in Austria and responsible for approximately 2 Mt CO_2_e GHG emissions. Although all GHG emissions in the paper, pulp and printing sector are energy-related, the sector has a special position in the consideration of GHG emissions because the chemical pulping of wood produces black liquor as waste within the sector boundary. However, subsequently it becomes an energy carrier which is used in companies’ own CHP plants to generate electricity and heat. This peculiarity of the sector currently saves considerable amounts of externally purchased energy sources and must be considered in all climate neutrality considerations. Production activity is modelled based on an average annual production increase of 0.2 %/a, both for paper and pulp.

**Fig. 2:**
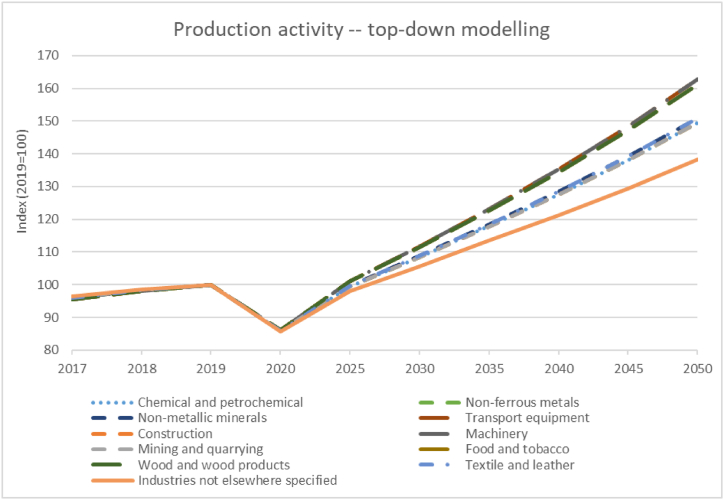
Assumed development of production activity for top-down modelling.

The remaining energy applications in the energy intensive subsectors and the remaining non-energy intensive subsectors are modelled top-down based on the projection of economic activity and the above-mentioned energy application categories – three temperature levels of heat, motive power, and lighting and information technology (IT). To project economic activity into the future, high-level studies on the economic development of Austria are used as a proxy for future production activity. The compound activity growth follows the adopted GDP growth over the study period. For robustness the model starts with historical GDP data from the period 2017 to 2019 with a total value of 360.14 billion €_2017_ in 2017 and growth rates of 2.4 % in 2018 and 1.6 % in 2019 [36,37]. For the following years, growth rates between −6.6 % in 2020 and 1.6 % after that until 2050 over the years and subsectors were used on average, varying only slightly between sectors [38,39]. The subsector-resolved results for the development of economic activity are presented in Fig. 2 (cf. Table A 2 in the appendix for absolute values 2019). Food and beverages, as well as wood and the transport and machinery subsectors are modelled with the highest increase in production activity. Heavy industries, such as construction, mining, or non-ferrous metals, as well as the top-down considered areas of the chemical and petrochemical industries are assumed in the midfield with industries not elsewhere specified trailing with the lowest activity increase.

Subsequently, the respective GDP data is translated into energy demands via sector-specific energy intensities as presented in Table A 3 to Table A 5 in the appendix. This approach takes into account yearly efficiency gains, differed by application category – thermal, motive power, and lighting/IT – and scenario.

### 2.2 Energy consumption scenarios

Three scenarios for future industrial technology deployment and resulting energy consumption are developed and calculated based on three distinct storylines as outlined below and also described in the report of the NEFI – New Energy for Industry project [30]. To guarantee applicability of chosen processes and technologies, respectively, scenario development was performed in a continuous feedback loop with industrial stakeholders. Note that the chosen scenario set differs from the set of above-mentioned existing transformation studies who follow a narrative set within the forcefield between the use of electrons vs. molecules as energy carriers [25]. We choose a different approach. Starting off from a baseline scenario to serve as a statistical reference point into the future, the two transitional scenarios aim to contrast current ambitions by key stakeholders within manufacturing industries against one technologically balanced deep decarbonisation scenario.

The scenario **Business as usual (BAU)** represents a trend scenario in accordance with the methodology put forth for such scenarios by Ducot and Lubben [40]. It serves as a reference scenario, allowing the evaluation of the effectiveness of innovative technologies in the two remaining scenarios. BAU is obtained by extrapolating historic statistical trends on the deployment rate of technologies within manufacturing industries and economic development forecasts. Announced but not yet implemented projects as well as any policy measures that have not already had a significant effect on past energy and emission statistics are not reflected in this approach. This puts the scenario in contrast to a “with existing measures” (WEM) scenario which would include forecasts based on current policies.

The scenario **Pathway of industry (POI)** represents a foresight scenario composed by a methodology as proposed by Martin [41]. It is the result of a close dialogue with technology officers from representative companies from all thirteen investigated subsectors, who have provided their plans and assessments of the technology deployments in their respective subsectors under current and foreseeable boundary conditions through to 2030. Development to 2050 is extrapolated on this basis and considers expected technology readiness. This extrapolation emerges from a tightly knit, workshop-based collaborative process, further involving the above-mentioned stakeholders. Initially, we formulated proposals based on preliminary studies (e.g. Rahnama Mobarakeh and Kienberger [34,42]) assessing the suitability of technologies for achieving climate neutrality. Subsequently, these proposals were synthesised in conjunction with the stakeholders to chart the course of further development up until 2050. The collaborative nature of this process ensures a comprehensive and holistic perspective, incorporating both scientific expertise and stakeholder input. The resulting scenario serves as a valuable framework for understanding and planning the trajectory towards climate neutrality in the coming decades. It is a unique and innovative representation of current industrial transformation plans in Austria and therefore well equipped to identify important areas of policy action in efforts to achieve climate neutrality. It thereby goes well beyond established stakeholder consultations known from existing scenario studies.

The scenario **Zero emission (ZEM)** is obtained by a backcasting approach as proposed by Robinson [43] to reach climate neutrality in 2050. This means that, starting from the target state of widespread adoption of deep decarbonisation technologies identified by Rahnama Mobarakeh and Kienberger [34,42] for the energy intensive industries and BAT-documents for general energy application in non-energy intensive subsectors (e.g., on energy efficiency [44]), a reverse pathway is developed indicating the steps leading to the successful achievement of the goal of far-reaching climate neutrality. Due to the chosen methodology of taking into account emissions of the upstream energy generation for industrial activity, it is important to note that complete industrial climate neutrality may not be achieved just through the modelled technology deployment. Nevertheless, the scenario represents the implementation of extensive and ambitious measures that can transform Austria's industrial energy system. Table 3 presents an overview of a selection of considered technologies, their assumed current technology readiness level (TRL) and the point of market entry modelled in scenario ZEM which were chosen from the above-mentioned subsector briefings and technology assessments. Due to the modelling timesteps of 5 years, the earliest possible market entry date for any future technology is 2025. The years in between the considered timesteps where deployment may begin to occur are not depicted. In the case of low temperature heat pumps, direct electric heating, electric engines, and other already existing low-emission technologies (e.g., solar thermal or district heating), deployment is modelled starting with the base year.

**Table 3:** Applied BTT by subsector in scenario ZEM.

Industrial subsector	Applied technologies	Current TRL	Start of scenario deployment
Iron and steel	Primary steelmaking by H-DR-EAF	7	2030
Chemical and petrochemical	H_2_-based primary production of methanol and olefins Biomass-based primary production of methanol and olefins H_2_-based ammonia production	8 8 8	2030 2030 2030
Non-metallic minerals	Carbon capture of geogenic emissions by oxyfuel technology implementation	6–7	2025
Paper, pulp and print	Extensive heat pump application for temperatures up to 200 °C Black liquor use in integrated mills with CHP plants	7 9	2025 Deployed
All subsectors (selected technologies)	Extensive electrification by low (LT) and high temp. (HT) heat pumps Direct electric heating Electric engines	LT: 9 HT: 7 9 9	LT: Deployed HT: 2025 Deployed Deployed

It is important to stress here once again, that the resulting scenario demands are not limited by additional framework conditions regarding the availability of energy or enabling infrastructure – except for scenario POI where these are reflected in industrial stakeholders' feedback.

### 2.3 Calculation of GHG emissions

Once energy consumption based on deployed technologies and type of energy carrier (e.g., electricity versus gas) is calculated based on the three scenarios, the energy consumption results are expanded with specific emission factors for energy-related, process-related and upstream emissions. In combination with the industry-focused measures considered for processes (e.g., novel process chains for steelmaking or in the chemical industry) and technologies (e.g., heat pumps or carbon capture technologies, among others) in already existing processes, a widely climate neutral supply side is essential if the manufacturing industries are to decarbonise. This includes, in particular, renewable electricity and gases as infrastructure-bound energy carriers. In the gas sector especially, changes in in-grid gas composition can affect the energy-related emissions intensity of the aforementioned energy consumption results. The applied methodology for the infrastructure-bound energy carriers is presented in 2.3.1 Gas grid and 2.3.2 Electricity grid.

In European industry, approximately 42 %^1^ of direct industrial GHG emissions are caused by process-related emissions, e.g. from primary steelmaking in blast furnaces or the use of carbonaceous materials such as CaCO_3_ in the non-metallic minerals sector [5]. Therefore, in addition to the consideration of energy-related emissions, their consideration is a centrepiece of the here-presented scenario development. For their calculation, specific emission factors per production technology were applied on subsectoral and process level in t CO_2_/t of product output. The considered subsectors include iron and steel, the chemical and petrochemical industry, non-metallic minerals, and paper and pulp.

Emissions from the incineration of carbonaceous energy carriers in final energy applications are calculated according to official specific emission coefficients as published by the Austrian environmental agency (UBA) [28]. For emissions from gas combustion, our methodology offers a novel approach to calculation of industrial emissions of the previously calculated demand for chemically stored gaseous energy.

#### 2.3.1 Gas grid

Where industries are supplied with energy via the gas grid, the in-grid gas composition varies due to larger developments within the overall energy system in the considered time frame to 2050. Therefore, the current exclusively CH_4_-transporting gas grid is gradually transporting a more and more diverse mix of fossil CH_4_, bio-CH_4_ and hydrogen. We have applied a separate methodology for modelling the in-grid gas composition in the three scenarios as outlined in the corresponding project report [30] and summarised below. Thereafter, the above-mentioned emission intensities are applied to the respective fossil CH_4_ content where necessary. Upstream emissions from biomethane are excluded. As Majer et al. [45] point out, the GHG mitigation potential of biomethane in comparison to its fossil counterpart varies depending on the production pathway between 51 % when utilising maize silage and 202 % when production is slurry-based which generates its high mitigation potential from avoiding emissions from untreated slurry.

The evolution of the overall gas supply system's composition is driven by increasing CO_2_-costs and decreasing costs for electrolysis production of hydrogen due to learning and scaling effects. To adequately model the available quantities of bio-CH_4_, fossil CH_4_ and hydrogen, a cost-based methodology to assess the composition of the Austrian gas grid in scenarios POI and ZEM was chosen. While in BAU industry-focused technology deployment and therefore also resulting energy consumption follows the statistical extrapolation approach explained in section 2.2, the available in-grid gas composition for final energy consumption is modelled in accordance with current national government targets for 2030 and their linear extrapolation until 2050. In this case 56 % fossil CH_4_ (energy share) remains in the overall gas supply system without consideration of costs.

In scenarios POI and ZEM, renewable gases reach cost parity with fossil CH_4_ between 2035 (ZEM) and 2045 (POI) and fossil gas is phased out. This means that in contrary to BAU scenario, the overall gas supply system does not contain any fossil CH_4_ from these points onwards. The admixture of hydrogen and bio-CH_4_ in the gas system from the year 2025 represents a transitional path with focus on reaching climate neutrality of the gas system by the time of the respective break-even point (BEP). It should be noted that the BEPs do not serve as specific input for dedicated H_2_-grids, but as an indicator that these become more and more widespread as we reach the BEP.

The considered carbon price development considers the price for carbon emissions which has been charged starting in July 2022 as part of the Austrian eco-social tax reform. As Table 4 visualises, the CO_2_ tax is set to be 30 €_2017_/CO_2_ in 2022 and 55 €_2017_/t CO_2_ in 2025 [46]. The further development of the CO_2_ prices corresponds to the assumptions according to the EU commission's guidelines for scenario WEM 2017 (for POI) and scenario Transition 2017 of UBA for ZEM [47].

**Table 4:** Assumed CO_2_ prices in €/t CO_2_e until 2050 in scenarios POI and ZEM [47].

	2025	2030	2035	2040	2045	2050
**POI [€/t CO**_**2**_**e]**	55	76	94	112	155	198
**ZEM [€/t CO**_**2**_**e]**	55	85	111	138	203	268

To calculate the available shares of each gas, *total gas consumption* for Austria is modelled in the first step. For the industrial sector, the herein-modelled industry scenario results – both for bottom-up manufacturing and top-down calculations – are used, while all other sectors (e.g., buildings and transport) are covered using the above-mentioned reports prepared by the Austrian environmental agency.

To merge the gas system modelling with bottom-up industry sector results (e.g., in iron and steel and chemical industry) in the second step, already defined – i.e., technologically required – gas types in these sectors (e.g., H_2_ in iron and steel) are subtracted from the overall gas system results. The process to calculate remaining energy amounts per gas type is visualised by eq. (1), with *t* representing each of the three distinct gas types, fossil CH_4_, bio-CH_4_, and H_2_, respectively.

(1) $$E_{t,Gas\;Grid,rem.}=T_{t,Total}-\;T_{t,Bottom-up}$$

**Fig. 3:**
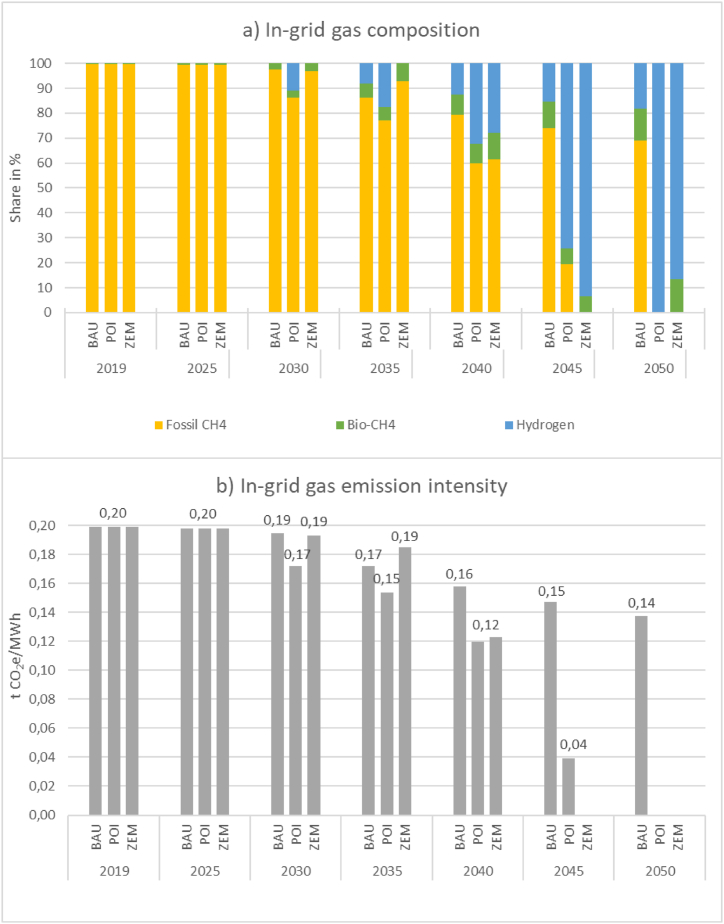
Considered in-grid gas composition (a) and resulting emission factors (b).

The remaining renewable gases comprise the in-grid gas mix visualised in Fig. 3, available to all users connected to it, i.e., also non-industrial users. This gas grid is a virtual representation of all physical grids that remain after subtraction of dedicated pipelines for the energy-intensive users already considered bottom-up. In scenario BAU, after subtraction of H_2_-consumption modelled in the iron and steel industry, extrapolation of government targets results in a 35 % share of renewable gases by 2050, of which 20 % points are provided by hydrogen. In scenario POI, the iron and steel industry and chemical and petrochemical industry rely predominantly on CH_4_-based production processes (cf. exemplary subsector results in the appendix). Therefore, by 2045, only hydrogen remains for supply to customers connected to the gas grid. In individual cases, the modelled in-grid gas composition means that if hydrogen from the overall gas grid is not suitable due to flame specifications, (carbonaceous) H_2_ derivatives must be produced on-site. In scenario ZEM, processes modelled bottom-up for iron and steel and the chemical and petrochemical industry rely more strongly on H_2_. Therefore, as bio-CH_4_ production ramps up, more and more CH_4_ becomes available as admixture in the predominantly H_2_-based gas grid.

#### 2.3.2 Electricity grid

**Fig. 4:**
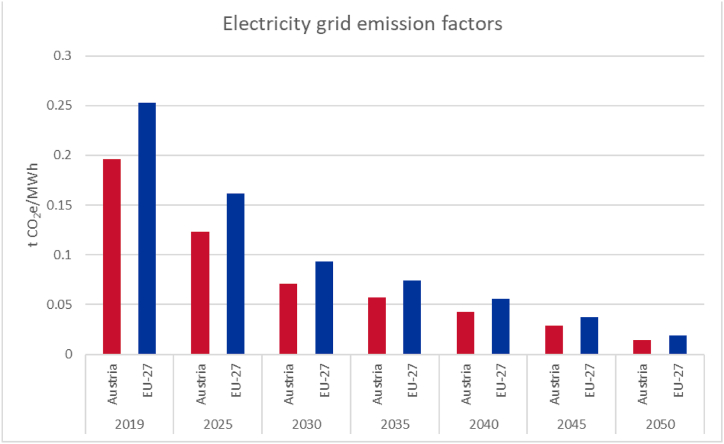
Assumed electricity grid emission factors development based on data from Ref. [50].

As mentioned in section 2.1 above and the corresponding project report [30], in our proposed methodology, we include the GHG emission intensity of upstream electricity generation to avoid merely moving the challenge of decarbonisation from one sector to another. Thereby, we are reflecting the interdependencies between industry decarbonisation and the Austrian electricity system as well as Austria's shared dependencies with the ENTSO-E network. To provide an estimate of the actual GHG intensity of industrial transformation, a decarbonisation path for the electricity system formulated by the European Commission for the EU-27 in Scenario MIX is used as basis [48]. Since the GHG-intensity of the Austrian electricity sector has historically been lower than that of the Union, we used the Austrian case as the starting point according to Ref. [49]. Thereafter, the European development as percentage from 2020 onwards is applied. The derived GHG emission development is illustrated in Fig. 4. Starting from 0,19 t CO_2_e/MWh of electricity consumed in 2019, the chosen approach results in a reduction of approximately 93 % to just 0.015 t CO_2_e/MWh by 2050.

## 3 Case study results and discussion

While we have modelled the Austrian manufacturing industry based on subsectors and in some cases as explained above on a manufacturing level within these subsectors, in this section only the aggregate of all investigated subsectors is presented and discussed to preserve conciseness. Exemplary subsector results for iron and steel, chemical and petrochemical, and non-metallic minerals where investigations on a manufacturing level are of special importance, as well as machinery as an example for non-energy intensive subsectors can be found in the appendix. The report of the project NEFI – New Energy for Industry in the course of which the methodology in this paper was developed, provides a collection of all subsectoral results [30].

**Fig. 5:**
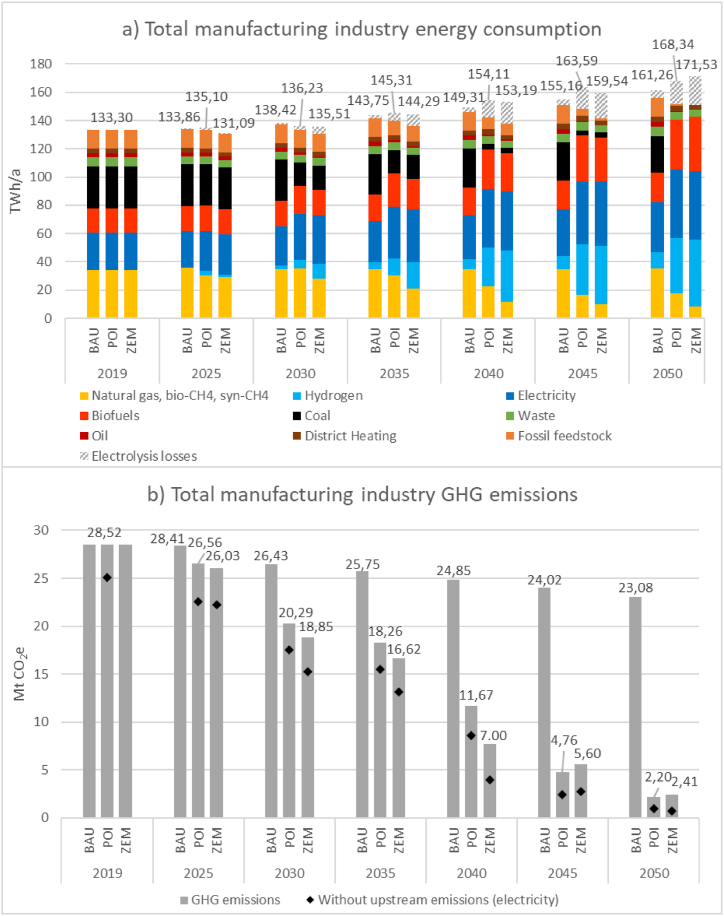
Total manufacturing industry results for a) energy consumption and b) GHG emissions for scenarios BAU, POI and ZEM.

Development for the aggregate of Austrian manufacturing industry is shown in Fig. 5. As visible, scenario BAU does not achieve meaningful GHG emission reductions compared to the base year. Due to assumed increasing economic activity, total energy consumption is further rising to up to 161 TWh (including 5 TWh of transformation losses for hydrogen production). Only the underlying decarbonisation trend in the electricity and gas grid does have a countereffect on emissions and can account for a reduction of approximately 5 Mt CO_2_e/a. In POI and ZEM on the other hand, fossil fuels with high emission intensities, most notably coal, oil, and fossil waste, are phased out and eventually replaced completely by less GHG-intensive or CO_2_-neutral alternatives. Emissions already decrease by approximately 35 % in both scenarios in the period between 2025 and 2035.

Total results in scenario POI indicate that, based on a technological approach, a GHG emission reduction of Austrian manufacturing industry larger than 92 % compared to 2019 until 2050 is possible if the transformation plans stated by industry representatives are taken into account and enabling conditions – especially in the form of a largely climate neutral energy supply system – are met. In comparison to scenario BAU, this is especially true for the gas grid where close to 60 TWh of largely climate neutral gases are needed by 2050. In the electricity grid on the other hand, further decarbonisation development until 2050 has a smaller impact due to already low emission intensities in the Austrian electricity sector. Most notably, widespread agreement exists among subsector representatives from different companies on the currently envisioned most important technology pathways and energy carriers for their respective subsectors. Total energy consumption in the scenario rises from 135 TWh to 151 TWh (168 TWh when electrolysis losses are included). Solid biomass consumption increases by 18 TWh from 17 TWh today to over 35 TWh by 2050. Approximately 48 TWh of final electricity consumption is projected by 2050 which signifies an increase of more than 22 TWh from 26 TWh in 2019. In the non-metallic minerals sector, over 3.7 Mt CO_2_e are captured by 2050. Some of this CO_2_ (1.8 Mt) could be utilised in the chemical industry to produce methanol and olefins. While this combination has not been further investigated in this study, it is apparent, that due to the resulting gap between CO_2_ supply and demand within the manufacturing industry, storage and export strategies for captured CO_2_ will be needed.

In scenario ZEM, the modelled technology deployment relies strongly on hydrogen, especially in the energy intensive subsectors iron and steel and chemical and petrochemical industry. By 2050, GHG emission mitigation of 92 % can be realised when including upstream electricity demand and associated emissions for hydrogen generation. Due to the largely decarbonised nature of all other energy carriers, these upstream emissions have a significant effect on the resulting emissions as visualised by the black diamonds in the figure. An especially large decrease of emissions can be observed between 2035 and 2040 due to both hydrogen reaching the BEP with fossil CH_4_ and large production volumes in iron and steel being moved from the BF/BOF route to H-DR-EAF. Total energy consumption rises to approximately 172 TWh by 2050, with more than 20 TWh from electrolysis losses already accounted for. Similarly to scenario POI above, solid biomass and electricity consumption both increase by approximately 20 TWh each in comparison to the base year. In comparison to POI, CO_2_ uptake by the chemical and petrochemical industry is higher due to the modelled deployment rate of H_2_-based methanol synthesis. By 2050, 2.6 Mt of CO_2_ are used in chemical production processes. In comparison to 3.70 Mt CO_2_ captured in the non-metallic minerals, the annual demand for net storage or export capacities is reduced to approximately 1.1 Mt CO_2_.

**Fig. 6:**
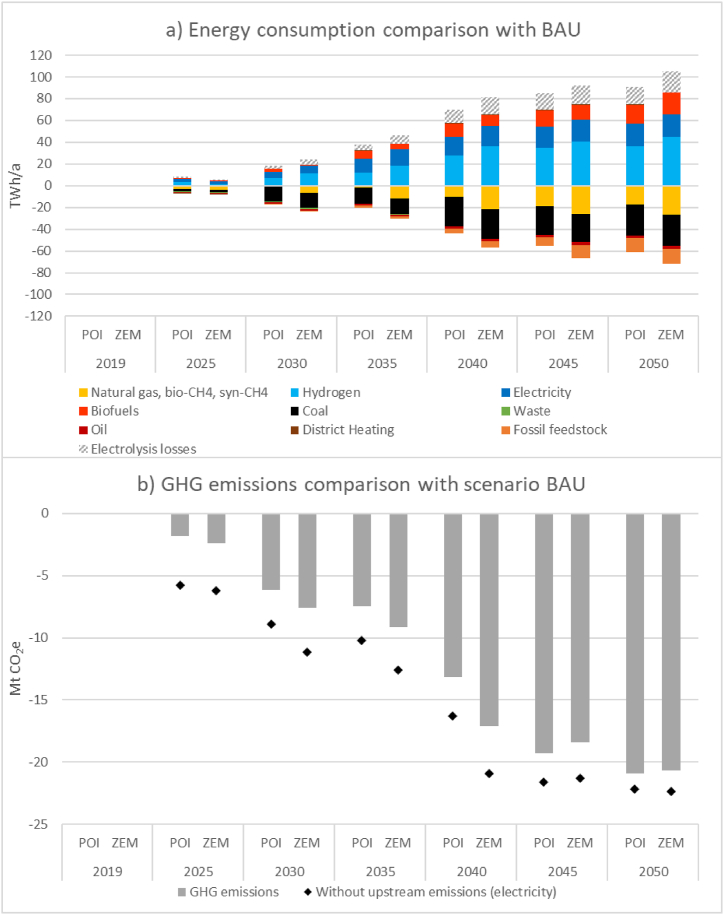
Total manufacturing industry results for a) energy consumption and b) GHG emissions for scenarios POI and ZEM visualised as difference to scenario BAU.

Fig. 6 presents another angle of investigation by showing the difference in results for scenarios POI and ZEM as a delta in comparison to the baseline scenario BAU. The bottom diagram represents GHG emission deltas, while the top diagram shows resulting deltas for total industrial energy consumption. Until 2025, the total energy consumption and GHG emissions in POI and ZEM scenarios largely follow the BAU scenario. From 2025 onwards, POI and ZEM exhibit differing technological pathways from BAU, resulting in contracting GHG emissions and a differing structure of energy carriers. As evident from comparing the results for scenarios POI and ZEM, Austrian industry representatives by-large already do envision a low emission pathway for their respective subsectors. While relying on different technologies, resulting energy consumption and GHG emissions in the two scenarios over the aggregate of subsectors are actually very similar. However, as exemplary visualised in the appendix, the subsector results for energy carriers can differ significantly between POI and ZEM due to different technology and thus also energy carrier deployment.

In both transition scenarios, energy consumption is characterised by three basic forms of energy carriers – electricity, gases, and biomass – while in BAU the energy mix is significantly more diverse because of the use of several mostly fossil energy carriers both for energy and non-energy use (e.g., coal, naphtha, oil). To substitute these energy sources, extended production chains are necessary which exhibit greater transformation losses (e.g., H_2_ from electrolysis for the non-energy use in the chemical sector, see appendix). Therefore, when considering electrolysis losses, slightly larger total energy consumption can be seen for the POI and ZEM transformation scenarios than for the reference scenario BAU (161 TWh) where upstream energy losses of fossil fuel production are not taken into account. The difference in necessary upstream production chains is also the reason for slightly higher ZEM results for total energy consumption (172 TWh) than can be observed for POI (167 TWh). As ZEM requires greater amounts of hydrogen, more transformation losses for electrolysis occur. This does not only affect the development of energy demand but also that of the associated GHG emissions. Beginning after 2040, when the gas grid in POI has reached the BEP of hydrogen and is thereby largely climate neutral, total GHG mitigation in POI in comparison to BAU is higher than in scenario ZEM. However, if the hatched area of electrolysis losses that may also be situated upstream inside the energy industries, both domestically and abroad, is disregarded, scenario ZEM results in slightly lower energy consumption than POI. The same is true for GHG emissions – without upstream emissions considered, instead of trailing by 0.2 Mt, scenario ZEM leads POI in its mitigation results in comparison to BAU by approximately the same number.

The above-described results, and especially the shown dependencies of manufacturing industries on largely decarbonised upstream energy provision show that large amounts of renewable energy sources (RES) must be made available to Austrian industry. As noted above, except for scenario POI where industry representatives’ assessment of framework conditions implicitly also includes availability of energy sources and infrastructure, no additional limits to energy supply have been considered in the presented demand scenarios. For example, both in scenarios POI and ZEM, 2050 biomass consumption almost doubles in comparison to BAU to up to 38 TWh (ZEM). For electricity applications, approximately 50 TWh of electrical energy for final energy applications is necessary in ZEM; in POI, this category amounts to 35 TWh. Taking into account current total Austrian electricity consumption in 2022 of approximately 64 TWh [27], the magnitude of the challenge of climate neutrality in industry becomes apparent. Current development speed of Austrian RES generation may not be able to fulfil this need. The modelled consumption for 2050 of manufacturing industries alone surpasses the amount Austria has set out to install in renewable electricity generation between 2018 and 2030 (27 TWh), highlighting the need for further efforts in this regard – already now but also beyond 2030 [51]. Besides general electrification efforts, e.g., heat pumps, direct electric heating and for motive power, the electricity consumption is especially driven by the decarbonisation of process-emission intensive subsectors such as iron and steel, the chemical and petrochemical industries, and non-metallic minerals. In these subsectors, the introduction of electric arc furnaces and possibly subsector-overarching carbon capture and utilisation processes signifies an important additional demand. Breakthrough technologies for carbon capture, e.g., oxyfuel, can reduce this amount to a necessary minimum while alternative technologies that are currently more economically viable, have a higher energy demand.

The extensive electrification efforts by use of heat pumps and electric engines also have an important impact on the overall gas consumption. Until 2050, consumption of gases only increases by approximately 10–15 TWh. Most notably, use of gases is shifted from final energy consumption for low or medium temperature and motive power applications to more exergetically-valued deployment as reducing agent and feedstock for non-energy use in basic material production in the chemical and petrochemical industry and primary steelmaking. Overall, approximately 50 TWh (POI) to 64 TWh (ZEM) of climate neutral gases are needed. The applied methodology for the gas grid composition shows cost leadership of renewable gases (mostly H_2_) due to rising costs of CO_2_ emission certificates and expectable electrolysis learning and scaling benefits. The combination with the modelled industrial gas consumption exhibits the immense impact and importance of a largely decarbonised gas grid on attaining deep-reaching steps towards climate neutrality in manufacturing industries.

The transition to climate neutrality will rely on the ability of national economies to provide the necessary amounts of energy. Therefore, the industry scenario results developed with energy demand in focus cannot be viewed in isolation but in consideration of the overall energy consumption and supply system. Taking into account the possible additional electricity needs for hydrogen production via electrolysis, total electricity consumption for industrial production in Austria rises from 26 TWh in 2019 to approximately 104 TWh in POI and 116 TWh in ZEM. Sejkora et al. [52] have calculated *technical* potentials of renewable energy sources in Austria to amount to approximately 266 TWh. In contrast, sector-resolved investigation of the most exergetically efficient way of supplying the useful exergy demand in total energy system analyses based on additional investigations by Sejkora et al. [53] reveal up to 41 TWh of electricity consumption and additional 36 TWh of hydrogen demand in the remaining economic sectors (i.e. transport, buildings, agriculture) in Austria. As not all technical potentials can be realised or are economical to use on the path to climate neutrality, a significant import share is highly likely in this case. In light of these limited domestic renewable resources, the consumption scenarios can show decision makers the need to also take into account alternative energy supply, e.g., by means of suitable and reliable import routes and the enabling infrastructure.

The above-discussed developments show that the industrial energy system of the future can operate at almost net zero emissions with the widespread use of three dominating key levers; electrification and general energy efficiency measures, carbon neutral gases and biomass utilisation, and carbon capture and usage as well as storage. Other technology solutions, such as solar thermal, wide-spread high-temperature electric direct heat, alternative binders for cement production or the deployment of complex bio refinery structures are not necessary according to both industrial stakeholders (scenario POI) and previous scientific investigations modelled by way of scenario ZEM (cf. investigations on industrial climate neutrality by Rahnama Mobarakeh and Kienberger [34,42], Fais et al. [54] and Johannsen et al. [55]).

## 4 Conclusion

As an especially emission-intensive economic sector but equally important backbone of national wellbeing, the manufacturing industries represent one of the key challenges towards climate neutrality for developed countries. For a successful transition, decision makers both on a political as well as an industrial level, must be able to assess the impact of their measures and actions. Scenario development can play a vital role in this process if able to provide both the necessary level of subsectoral detail *and* a broad understanding of interrelations – among industry subsectors, as well as in relation to the overall energy system where emissions from industrial activity are also dependent on upstream decarbonisation efforts.

When using energy consumption scenarios for long term decision and policymaking regarding technology deployment in manufacturing industries, it is important to link the modelled energy demands with the supply side of energy carriers. As we have seen, while the modelled technologies strongly differ between scenarios POI and ZEM, resulting emissions show much smaller differences – due to the chosen methodology for hydrogen shares in the gas system and the upstream electricity generation. Most strikingly, the results of scenario POI until 2050 show that Austrian manufacturing industries already envision a very progressive technology deployment which comes very close to climate neutrality when assuming a largely climate neutral supply side for gas and electricity. In comparison to the envisioned deep decarbonisation scenario ZEM which is prepared using a balanced deployment of best available and breakthrough technologies, less emphasis is put on energy efficiency in final energy application, and less hydrogen availability is expected. The resulting similarities in aggregate results of the two transition scenarios POI and ZEM underline important areas of action to provide framework conditions to enable far-reaching decarbonisation. Thereby we can raise the relevance of the scenario results for political stakeholders.

### 4.1 Limitations of current work

Certain areas remain, where this line of research could be further strengthened which we want to highlight. It is of special importance to note that, for successful implementation of the shown methodology, extensive data availability, both from official statistics and industrial stakeholders is key to modelling the investigated industrial system. The presented scenarios incorporate all energy-related units within the thirteen subsectors of manufacturing industries. However, as the approach proposed by the authors in Ref. [31] has not been adopted by official statistics yet, necessary data on subsector level had to be rebuilt from higher aggregation levels, leaving space for uncertainty. Availability of industrial data on consumption aggregates and subsectoral or production plant use of energy transformation units, especially CHP plants, is therefore a current limit and presents great improvement potential for future studies.

In addition, several areas of our chosen approach merit additional discussion and could be taken up in subsequent investigations. Firstly, as noted in the introduction, from our point of view, the focus on the manufacturing industries alone – without an overarching energy system modelling to take into account the necessary upstream energy provision – is better suited to direct the focus of industrial stakeholders and policymakers in the area of industrial policies on matters of technology research and enabling framework conditions. On the other hand, one must acknowledge that this approach must make use of less comprehensive upstream energy system analyses than total energy system reports that embed manufacturing industries as one of many economic sectors. The decision on what pathway to choose – focused on industry with more assumptions on the overall energy system or more subsector-resolved industrial energy system analysis – must be made with a deep understanding of the necessities of the target groups and stakeholders as advantages and disadvantages must be weighed against each other. For example, the assumptions we have made on the GHG intensity of the gas and electricity grids may very well be challenged by more holistic analyses. This includes the range of CO_2_ prices reflected in the in-grid gas composition, the neglection of (possibly negative) emissions along the biomethane value chain, as well as the single electricity grid model applied to all scenarios uniformly. The significance of these assumptions has been illustrated impressively by comparison of scenarios POI and ZEM by 2050. To remedy this issue within the proposed methodology, future sensitivity analyses will have to investigate the impact of a range of assumptions regarding the upstream energy generation processes.

Further investigations on climate neutrality pathways in manufacturing industries must take up additional technological focus areas, e.g., widespread adoption of circular economy measures, further electrification efforts also in the areas of high temperature heat, production chains that rely more strongly on e-fuels or investigate the possible impact of technologies with a currently lower TRL. Regarding the sequestration and subsequent use or storage of CO_2_, the here-presented study supplied only a basic CO_2_ balance where we put captured CO_2_ from non-metallic minerals side-by-side with possible CO_2_ sinks in the chemical industry. Precise analyses of further CO_2_ use in chemical production, future consumers' use and eventual disposal must be conducted in future publications. Based on the here-proposed scenario set, additional industrial representatives can further refine the results and expand the current focus which has been dominated by large key representatives to include more small and medium sized companies. It must also be noted here that scenario POI can be a very dynamic scenario as multiple factors can influence industries’ current assessment of future technology deployment. In all these cases, as well as for the existing scenario results, techno-economic analyses will help to further assess the need for enabling policymaking (e.g., funding or market regulation) and expanded R&D activities in industrial subsectors.

### 4.2 Summary and conclusion

The approach presented in this work aims to provide energy demand and GHG emission scenarios for the transformation of the manufacturing industries that can inform both decision making by industrial stakeholders on a subsectoral level *and* policymaking by virtue of the following three aspects.

1. Application of three distinct scenario narratives, taking into account stakeholder decarbonisation plans.

The applied scenarios allow for the investigation of a bandwidth of possible developments, beginning from trend extrapolation, ranging over assessments of already-planned investments and transformations from the point of view of key industrial representatives to the wide-spread use of scientifically identified breakthrough-technologies with the highest available efficiencies.

2. Use of subsectoral resolution for investigation and calculation of all processes related to the respective primary economic activity.

We calculate energy consumption and GHG emissions of all processes related to the respective primary economic activity, including industrial autoproducers, non-energy use and transformation units. This approach tries to find a happy medium between both a holistic view on the energy consumption of any given subsector on the one side and comparability of results between subsectors on the other.

3. Consideration of upstream energy provision and applicable GHG intensity.

We include a cost-driven investigation of the future in-grid gas composition to indicate the GHG intensity of the necessary gas supply. For electricity, Austria's embedding in the European energy market is considered. This inclusion of upstream GHG emission intensities and transformation input (e.g., electrolysis) allows the necessary investigation of industrial dependencies on the overall energy system. As noted above, it allows a compromise between an industry-centred detailed analysis and a more holistic investigation of the overall energy system of a country which we have discussed in the introduction.

Comparison of the developed scenarios allows for the identification of no-regret measures to enable climate neutrality in manufacturing industries' subsectors by 2050 as set forth by the European Commission – not only from a scientific and technological point of view, but very importantly also from the view of industrial stakeholders who already have certain transformation plans in place. We have identified these measures making one basic assumption: We assume these measures increase the attractiveness of the industrial location due to high technological development and an accompanying enabling infrastructure. Thereby, total cost of production via climate neutral energy and technologies remains but one factor in industries’ decision making on future locations of activity. To further investigate the framework conditions regarding the choice on future locations of production, the cost structure of herein identified large technological levers of action for climate neutrality in comparison to the currently deployed fossil base case must be assessed in future publications. For the Austrian case study [30], the following no-regret measures can be identified.

• To achieve climate neutrality, energy-intensive and non-intensive industries require different areas of focus as they stand at different stages of their transformation. Energy-intensive industries need to prioritise researching, developing, demonstrating, *and* rolling out their specific technologies to achieve their targets. On the other hand, the non-energy intensive subsectors need to put special emphasis on accelerating the implementation of already-existing cross-sectoral technologies such as heat pumps to maintain their competitive advantage and stay on track towards climate neutrality.
• For the fast implementation of new technologies in manufacturing industries, not only research and development but *also* demonstration is crucial. Therefore, intensified and accelerated efforts are necessary in both these areas.
• Technological, logistical as well as policy related solutions for CO_2_ as a feedstock and with regards to storage options need to be found quickly.
• Supply of renewable energy carriers (especially CO_2_-neutral gases and electricity) must be secured to enable industrial transition towards climate neutrality. In light of limited resources that we have discussed above, the utilisation of these energy carriers should be prioritised based on technological requirements as well as temperature or exergy levels (e.g., CO_2_-neutral gases for high-temperature processes and process demands, heating and cooling by heat pumps).
• Taking into account other economic sectors, Austria's gross domestic energy demand could surpass the technical potential for renewable energy sources in the country. Therefore, it is essential to develop import strategies, particularly for CO_2_-neutral gases and their derivatives, to ensure a sustainable and secure energy supply for the future.
• The energy infrastructure must be updated to align with the aforementioned developments. This includes expanding the capacity of domestic and cross-border electricity grids, as well as building infrastructure for hydrogen and its derivatives.

In conclusion, identified measures can relate to all parts of the energy system, ranging from energy generation and supply, over energy infrastructure, to energy use and the deployed process technologies. As seen in the case study, the model results can be used to derive recommendations on technology promotion needs, infrastructure developments and to identify possible corridors, focal points, and fuel shifts. The applied subsectoral focus makes the results relevant both on the level of subsector representatives and for high level policymakers. To identify infrastructural and import requirements, it is important to contrast resulting energy consumptions with existing regional potentials of RES. Thus, the developed energy consumption and GHG emission scenarios contribute to a better understanding of current and future necessities in the energy system and within subsectors.

## Funding statement

This work was carried out as part of the NEFI_Lab project. The NEFI_Lab project is supported with funds from the Austrian Climate and Energy Fund and implemented in the framework of the RTI-initiative “Flagship Region Energy”.

## Data availability statement

No data associated with the study has been deposited into a publicly available repository.

Data included in article/supp. material/referenced in article.

## CRediT authorship contribution statement

**P. Nagovnak:** Writing – review & editing, Writing – original draft, Visualization, Methodology, Investigation, Data curation, Conceptualization. **C. Schützenhofer:** Writing – original draft, Conceptualization. **M. Rahnama:** Investigation. **R. Cvetkovska:** Methodology. **S. Stortecky:** Investigation, Data curation. **A. Hainoun:** Methodology, Investigation, Data curation. **V. Alton:** Data curation. **T. Kienberger:** Writing – review & editing, Supervision, Methodology, Funding acquisition, Conceptualization.

## Declaration of competing interest

The authors declare that they have no known competing financial interests or personal relationships that could have appeared to influence the work reported in this paper.

## Figures and Tables

**Fig. A 1:**
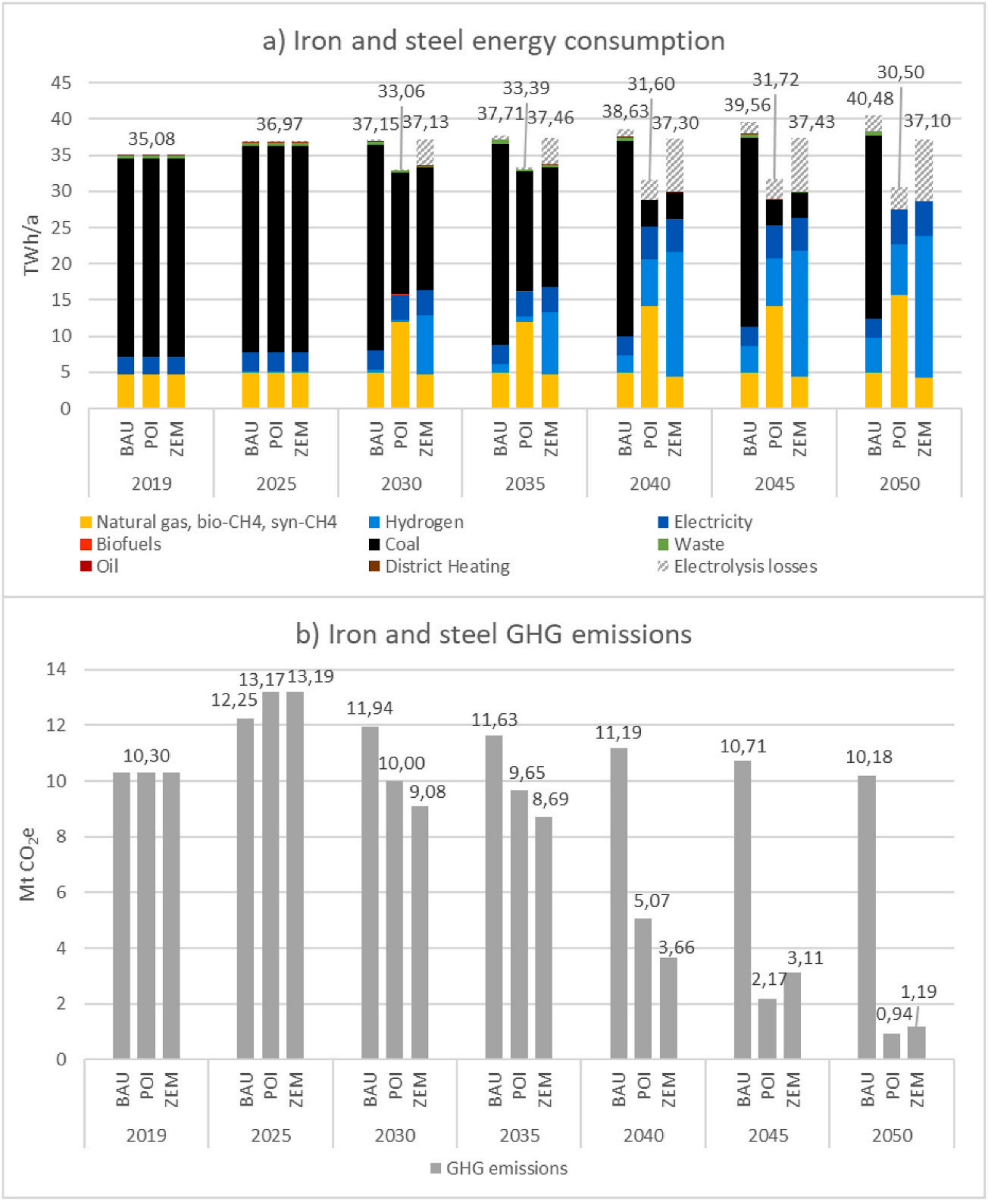
Iron and steel results for a) energy consumption and b) GHG emissions for scenarios BAU, POI and ZEM.

**Fig. A 2:**
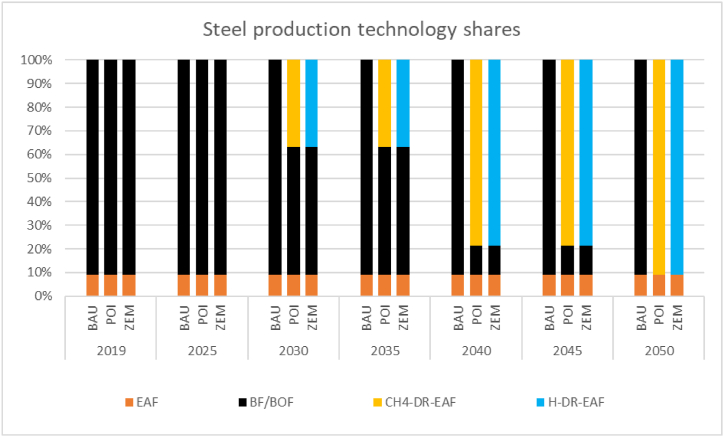
Development of technology shares in steel production in scenarios BAU, POI and ZEM.

**Fig. A 3:**
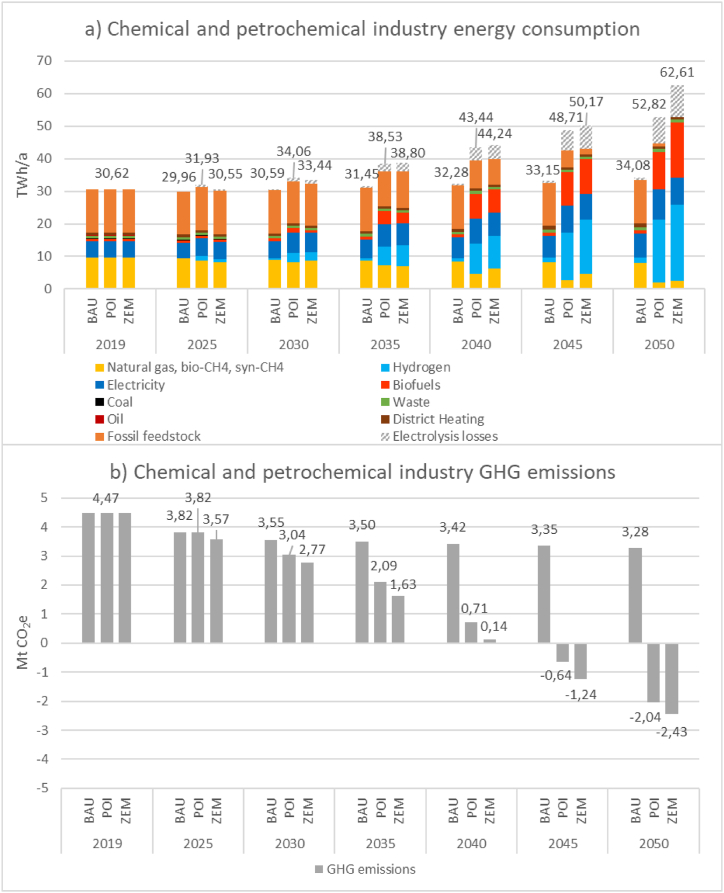
Chemical and petrochemical industry results for a) energy consumption and b) GHG emissions for scenarios BAU, POI and ZEM.

**Fig. A 4:**
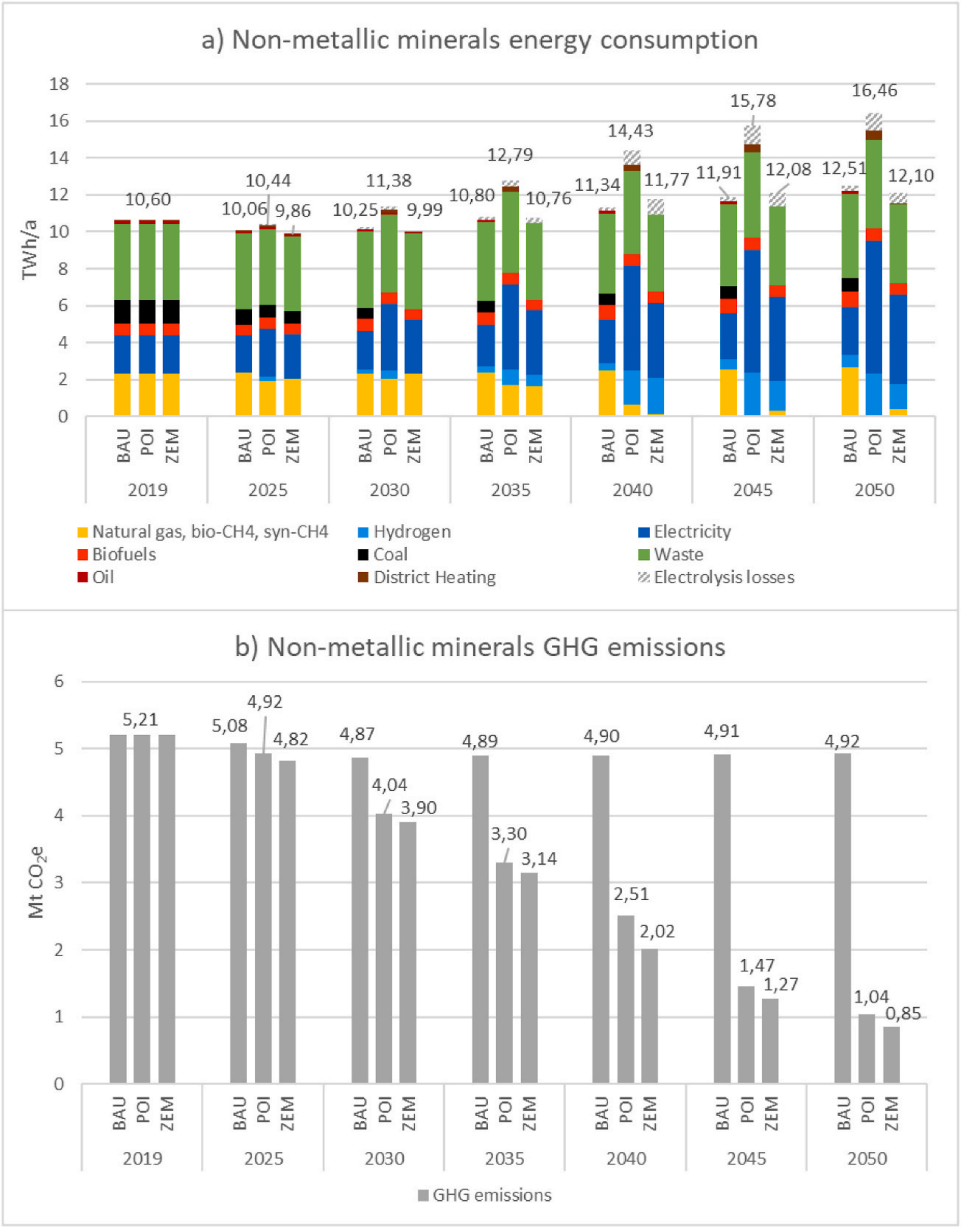
Non-metallic minerals results for a) energy consumption and b) GHG emissions for scenarios BAU, POI and ZEM.

**Fig. A 5:**
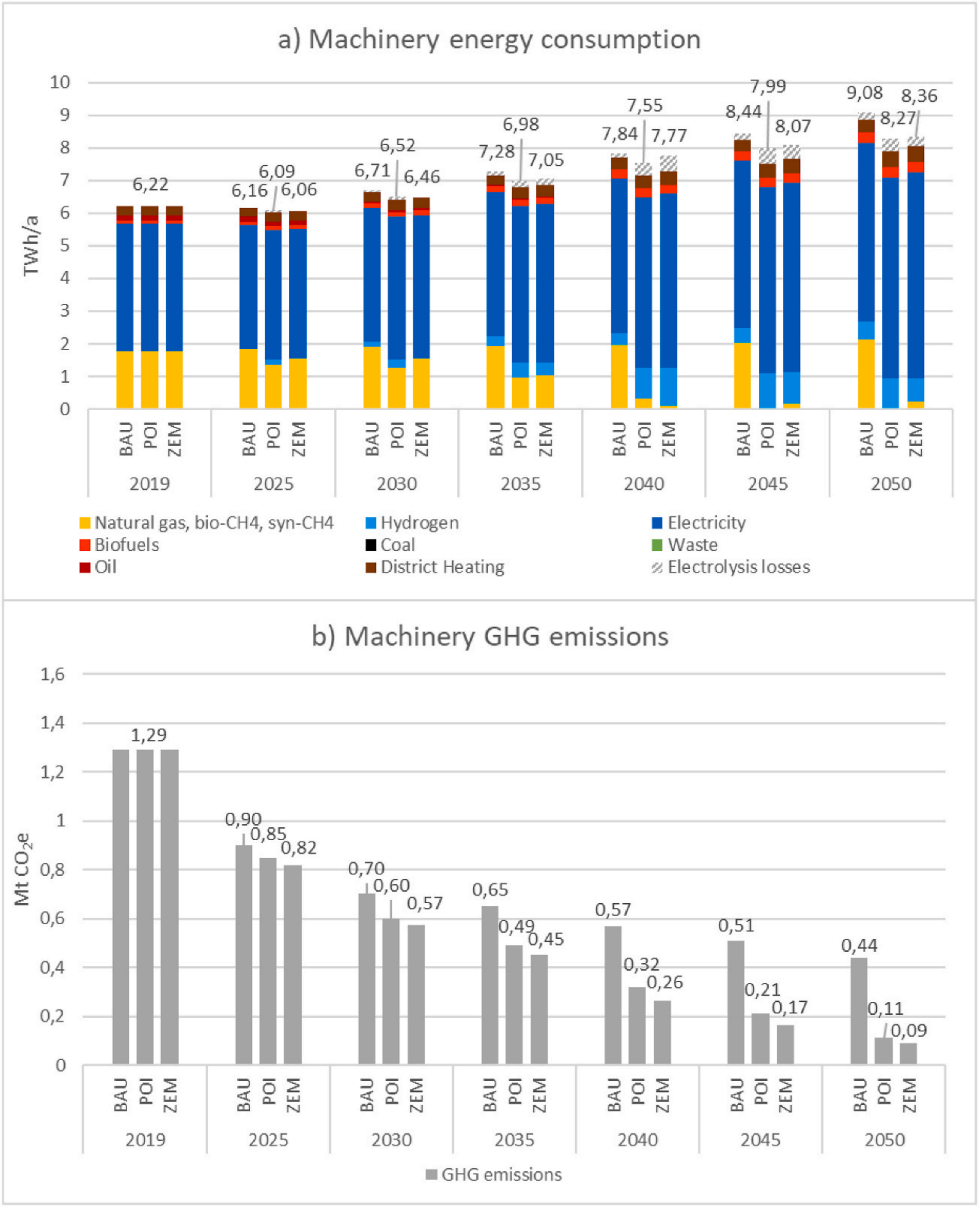
Machinery results for a) energy consumption and b) GHG emissions for scenarios BAU, POI and ZEM.

## APPENDIX

### Exemplary subsector results

Complementary to section 3, below we present exemplary results from chosen subsectors. As explained above, the report of the NEFI – New Energy for Industry project in the course of which this work has been carried out, includes detailed results of all thirteen investigated subsectors [30]. The first three subsectors – iron and steel, chemical and petrochemical industry, and non-metallic minerals – belong to the energy-intensive industries. As explained in section 2, in these subsectors the manufacturing units given in Table 2 are investigated and both transformation units and final energy consumption are considered. The finally presented subsector – machinery – serves as an exemplary result for the non-energy intensive industries where cross-sectional technologies such as heat pumps and direct electrification were modelled top-down in accordance with the methodology put forth in section 2.

### Iron and steel

The Austrian iron and steel industry is the sixth largest steel producer in Europe and in 2021 accounted for 5 % of European Union's (EU-27) steel production [56]. As can be seen in Figure A 1, in the scenarios' base year 2019, the iron and steel industry consumed in total approximately 35 TWh, corresponding to approximately 9 % of Austrian gross domestic energy consumption. Close to 90 % of this energy comes from fossil fuels, including coal, coke, natural gas, and bituminous coke. Fossil fuels are mainly used for two purposes: as a reducing agent (around 54 %) and as a source of energy to cover heating requirements. Approximately 10 TWh/a or ∼30 % of total energy consumption is used for final energy applications, primarily to provide high-temperature process heat in furnaces. Approximately 70 % are utilised in transformation processes in primary steelmaking (blast furnaces, coke ovens, etc.) [27]. Total annual CO_2_ emissions range between 10.3 Mt (2019) and 12.6 Mt CO_2_e (2017) per year, making the subsector an important contributor to Austria's overall emissions (approximately 14 % annually). The visualised increase in energy demand and GHG emissions between 2019 and 2025 results from the chosen production activity of a total of 8.1 Mt of steel (value of 2017, as shown in Table A 1) which as a conservative approach to activity development is kept constant from 2025 onwards in accordance with the communicated plans of the only Austrian company in primary steel production.

Figure A 2 presents an overview of modelled technology shares for steel production in the three scenarios. In all three scenarios, the share of secondary steelmaking via the EAF route has not been varied and remains constant at approximately 0.7 Mt/a, 10 % respectively.

Scenario BAU does not depict major changes in the mentioned fuel shares of total energy consumption. Reduction of iron ore is supplemented by injection of small shares of hydrogen into the blast furnace beginning in 2025 which are used to decrease the process-related emissions of the currently deployed production route (BF/BOF).^2^

On the other hand, the two transition scenarios POI and ZEM achieve very significant emission reductions of approximately 90 % compared to BAU by2050.^3^ The subsector's GHG emission results are most strongly influenced by the scenario-specific technology chosen for primary steelmaking, as well as the upstream emission intensity of electricity and gas supply. POI and ZEM especially differ in applied technology for primary steelmaking. Although CH_4_-based direct reduction (CH_4_-DR-EAF) is deployed in POI, the difference in GHG emissions remains small due to the modelled decarbonised nature of the gas supply system as described in section 2.3. In addition, hydrogen production through electrolysis drives emissions from the electricity sector's upstream emissions, resulting in higher total emissions in ZEM than in POI. By 2050, approximately 20 TWh of hydrogen are needed in scenario ZEM. This can generate electricity consumption via H_2_-electrolysis of more than 28 TWh/a of which transformation losses are presented by the shaded area on top of the bars in Figure A 1. The future location of these upstream energy consumption for hydrogen production may lie either partially or fully in Austria, Europe or, assuming adequate transportation infrastructure, anywhere in the world. In scenario POI on the other hand, the use of biomethane does not result in the same upstream energy losses in our methodology which is advantageous for POI in the shown results.

The greatest decrease in emissions from one 5-year period to the next is caused in the periods 2025–2030 and 2035–2040 by moving approximately 3 Mt of primary steel production each from the BF/BOF route to the DR/EAF technology. In POI, industrial stakeholders envision CH_4_-based direct reduction as an already-available technology with an auxiliary share of 30 % H_2_ in 2050. On the other hand, in ZEM, BTT by way of H-DR-EAF is deployed.^4^ The substitution of traditional BF/BOF production routes in both scenarios starts during the period 2025–2030 and reaches completion after 2045. By 2050, energy consumption in primary steelmaking in both POI and ZEM is exclusively characterised by three energy carriers: CH_4_, H_2_, and electricity.

In secondary metallurgy, the increasing use of BAT in all aspects of production causes a slight decrease in energy consumption in POI. The same trend can be observed in scenario ZEM, though slightly stronger due to more progressive electrification efforts.

### Chemical and petrochemical industry

Figure A 3 presents the three scenarios’ results for the chemical and petrochemical industry. To replace fossil feedstock (naphtha), which is currently non-energy use for producing olefins, in scenarios POI and ZEM, a higher increase in methanol production (for the methanol-to-olefins route) is assumed than in scenario BAU. Therefore, by absorbing up to 4 Mt of CO_2_ to produce methanol, the chemical industry becomes a net emission sink with −2 Mt and −4.2 Mt CO_2_e in scenarios POI and ZEM, respectively. With reference to the applied balance border discussed in section 2.1, it must be noted that in the case of the subsector investigation of the chemical and petrochemical industries, neither the origin of the captured CO_2_ nor the final disposal of products from the subsector are taken into consideration which is why negative emissions can be achieved in the subsector results but not in the overall manufacturing industries shown in section 3.

The difference of about 2.1 Mt CO_2_e in GHG emissions between scenarios POI and ZEM originates from the complete change of the technologies to a hydrogen-based low carbon route of olefin production in ZEM. The effects of replacing the currently imported and energy-dense feedstock naphtha (non-energy use, shown as “fossil feedstock” in the figure above) with methanol by domestically synthesising it from hydrogen and CO_2_, increases total energy consumption of the chemical subsector significantly by 73 % in scenario POI and over 100 % in scenario ZEM. The specific emissions change here in the largest product range from 0.91 t CO_2_e/t_olefins_ (naphtha sourced) to 0.36 t CO_2_e/t_olefins_ when methanol is the feedstock. Transforming methanol production using biomass or hydrogen and CO_2_ as feedstock, changes specific emissions from 0.55 t CO_2_e/t_methanol_ to −1.37 t CO_2_e/t_methanol_. As we have seen for iron and steel above, here too, the large hydrogen consumption generates a strong impact on the upstream energy provision. By 2050, 20 to 23 TWh of H_2_ are needed in the subsector alone. This can generate electricity consumption via electrolysis of more than 33 TWh/a. However, the deployment of hydrogen for the production of methanol and its derivatives can offset the occurring upstream emissions from electrolysis which leads to a significant CO_2_ sink of 2.6 Mt CO_2_ by 2050 in scenario ZEM. This notable resulting forcefield between primary energy demand and emission sink must be taken into account when choosing the future production technologies in the subsector.

### Non-metallic minerals

In 2019, the subsector consumed approximately 10.6 TWh of final energy as visualised in Figure A 4. In contrast to the iron and steel or chemical industry, no energy transformation units are deployed in this subsector. The main energy carriers are fossil fuels (51 %) with high shares of natural gas, electricity (24 %) and alternative waste fuels (AWFs, 22 %) [27]. AWFs such as waste tires, and waste oil are currently used especially in the cement production.

In scenario BAU, the challenge of reaching climate neutrality in the subsector remains unsolved, both regarding process-related emissions and deployed fuels. The share of the fossil energy carriers – especially coal and oil – remains high.

Between scenarios POI and ZEM, the deployed technologies for carbon capture present the largest difference.^5^ In POI, industry stakeholders report the introduction of carbon capture using readily available amine scrubbing technology, incrementally starting from 2025 onwards. This allows the sector to reduce emissions by up to 83 % in comparison with the base year, sequestrating 3.70 Mt CO_2_ by 2050. Due to the deployed carbon capture options' matching CO_2_ sequestration rates of 90 %, direct GHG emissions from the subsector do not differ significantly between scenarios POI and ZEM. Staying strictly within the subsector's balance border, after sequestration only storage can be assumed. Additional downstream energy demand for the potentially necessary transport is not accounted for.

In scenario POI, heat consumption of the amine scrubber is supplied by the process' flue gas and the use of heat pumps. The high energy intensity of the chosen technology adds approximately 7 TWh of electrical energy to the subsector's consumption. In comparison, the technology driven ZEM scenario allows for a significantly reduced energy consumption due to deployment of oxyfuel technology instead of the amine scrubbing for carbon capture. For high-temperature applications prevalent in production processes of the subsector, decrease in total gas consumption cannot be observed in either scenario as gas is used as best available technology in accordance with investigations by Rahnama Mobarakeh and Kienberger [42]. In comparison to the base year (10.7 TWh/a), ZEM only shows an increase in energy consumption of 18 %, while scenario POI increases consumption significantly by 55 % to 16.5 TWh/a.

Only upstream emissions in the electricity sector account for a difference between the two transition scenarios. However, by 2050 the total difference amounts to just 2,2 % or 20 kt CO_2_e due to the highly decarbonised electricity grid in both scenarios.

### Machinery

The machinery subsector belongs to the non-energy intensive industrial subsectors. The subsector consists of a large number of small and medium sized companies and production sites. As it is widely comparable to the other non-energy intensive subsectors in Austria, we present it here as an exemplary case for the subsectors investigated top-down (cf. section 2).

As shown in Figure A 5, total annual energy consumption amounts to approximately 6 TWh. For the machinery subsector, an annual growth rate of 1.6 % is assumed. As visualised by Table A 3 to Table A 5 in the supplementary information section, efficiency gains for motive power, lighting/IT and thermal applications are in the same range as in other industrial subsectors. Hence, the overall energy consumption rises in all scenarios. Space heating together with low temperature process heat (up to 150 °C) represents the major energy application.

The low to medium temperature heat consumption up to 150 °C including for steam is modelled to be supplied by heat pumps with low diffusion rates in scenario BAU starting in 2040, and an accelerated roll out in POI and ZEM serving nearly all technically possible consumption in 2045. Apart from general growth, this trend is the main driver for decreased direct fuel consumptions and a strong rise in electricity consumption. The largest efficiency gains are therefore expected to come from providing space heating with heat pumps. In ZEM, thermal process energy consumption is electrified to a very large extent (above 150 °C using direct electric heating) from 2030 onwards. Hydrogen consumption presented is not process-specific but arises from the gas grid mix.

In the transition scenarios POI and ZEM, the machinery subsector is projected to reduce GHG emissions from a total of 1.3 Mt CO_2_e in 2019 to 0.1 Mt or by 92 % until 2050. The two scenarios mainly differ in the rates of technology diffusion in electrifying process heat consumption and the rollout of heat pumps for the medium temperature range, envisioned five to ten years later by industrial stakeholders in POI than modelled in ZEM.

## Footnotes

1 including emissions from product use (e.g., HFC) which is not investigated in the present study.
