# Supplementary material for: Assessment of technology-based options for climate neutrality in Austrian manufacturing industry
Source: Heliyon. 2024 Feb 1;10(3):e25382. doi: 10.1016/j.heliyon.2024.e25382 (PMC10864908; doi:10.1016/j.heliyon.2024.e25382)
Supplement: Multimedia component 1 [file mmc1.docx]

## Supplementary information


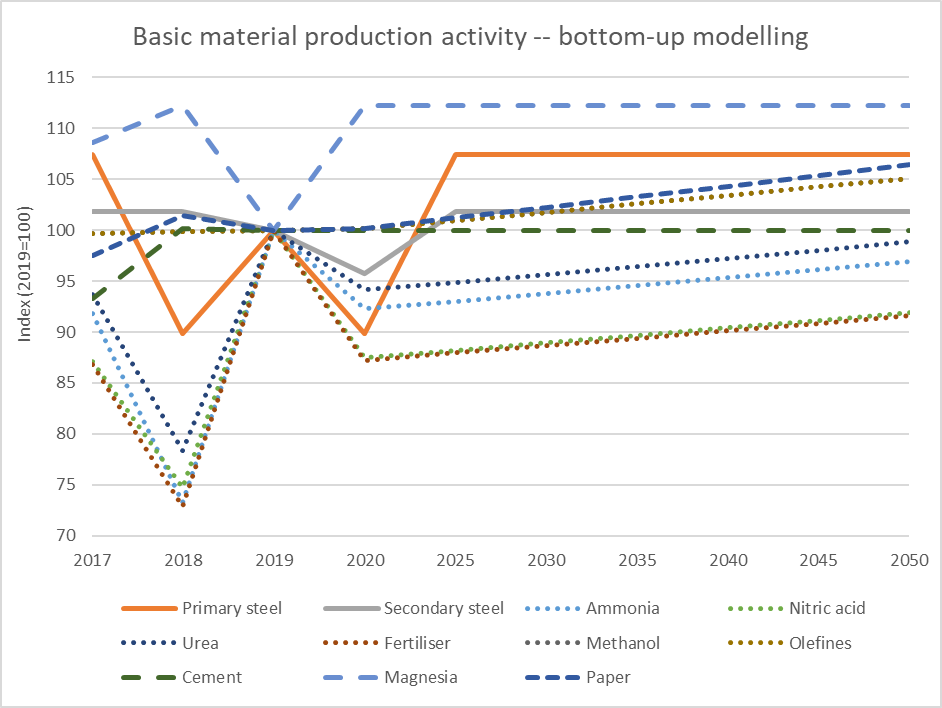


Figure A 6: Assumed development of production activity in basic material production.

Table A 1: Assumed production activity of basic materials in Mt/a.

| in Mt/a | 2017 | 2018 | 2019 | 2025 | 2030 | 2035 | 2040 | 2045 | 2050 |
| --- | --- | --- | --- | --- | --- | --- | --- | --- | --- |
| Primary steel | 7.41 | 6.20 | 6.90 | 7.41 | 7.41 | 7.41 | 7.41 | 7.41 | 7.41 |
| Secondary steel | 0.72 | 0.72 | 0.71 | 0.72 | 0.72 | 0.72 | 0.72 | 0.72 | 0.72 |
| Ammonia | 0.51 | 0.41 | 0.55 | 0.51 | 0.52 | 0.52 | 0.53 | 0.53 | 0.54 |
| Nitric acid | 0.50 | 0.43 | 0.58 | 0.51 | 0.51 | 0.52 | 0.52 | 0.52 | 0.53 |
| Urea | 0.42 | 0.35 | 0.45 | 0.43 | 0.43 | 0.43 | 0.44 | 0.44 | 0.44 |
| Fertiliser | 0.95 | 0.80 | 1.09 | 0.96 | 0.97 | 0.98 | 0.99 | 0.99 | 1.00 |
| Methanol | 0.03 | 0.03 | 0.03 | 0.03 | 0.03 | 0.03 | 0.03 | 0.03 | 0.03 |
| Olefins | 1.29 | 1.29 | 1.29 | 1.30 | 1.31 | 1.32 | 1.33 | 1.34 | 1.36 |
| Cement | 4.88 | 5.24 | 5.23 | 5.23 | 5.23 | 5.23 | 5.23 | 5.23 | 5.23 |
| Magnesia | 0.33 | 0.34 | 0.30 | 0.34 | 0.34 | 0.34 | 0.34 | 0.34 | 0.34 |
| Paper | 4.86 | 5.06 | 4.98 | 5.04 | 5.10 | 5.15 | 5.20 | 5.25 | 5.30 |

Table A 2: Development of production activity for top-down modelling in billion €_2019_.

| **in billion €_2019_** | **2017** | **2018** | **2019** | **2025** | **2030** | **2035** | **2040** | **2045** | **2050** |
| --- | --- | --- | --- | --- | --- | --- | --- | --- | --- |
| Chemical and petro-chemical | 5.80 | 5.95 | 6.04 | 6.02 | 6.55 | 7.12 | 7.71 | 8.34 | 9.03 |
| Non-ferrous metals | 1.59 | 1.63 | 1.65 | 1.64 | 1.79 | 1.95 | 2.11 | 2.28 | 2.47 |
| Non-metallic minerals | 2.89 | 2.96 | 3.01 | 3.01 | 3.28 | 3.57 | 3.87 | 4.20 | 4.55 |
| Transport equipment | 5.30 | 5.44 | 5.54 | 5.61 | 6.19 | 6.83 | 7.49 | 8.22 | 9.02 |
| Construction | 23.23 | 23.79 | 24.17 | 24.06 | 26.18 | 28.48 | 30.83 | 33.38 | 36.13 |
| Machinery | 26.39 | 27.10 | 27.61 | 27.95 | 30.83 | 34.01 | 37.32 | 40.96 | 44.94 |
| Mining and quarrying | 1.19 | 1.22 | 1.24 | 1.23 | 1.34 | 1.46 | 1.58 | 1.71 | 1.85 |
| Food and tobacco | 6.85 | 7.04 | 7.17 | 7.24 | 7.98 | 8.79 | 9.63 | 10.55 | 11.56 |
| Wood and wood products | 2.74 | 2.82 | 2.87 | 2.90 | 3.19 | 3.52 | 3.85 | 4.22 | 4.63 |
| Textile and leather | 1.10 | 1.12 | 1.14 | 1.14 | 1.24 | 1.35 | 1.47 | 1.59 | 1.73 |
| Industries not elsewhere specified | 5.84 | 5.97 | 6.05 | 5.94 | 6.39 | 6.86 | 7.34 | 7.84 | 8.37 |

Table A 3: Applied specific energy demand for motive power.

| **Specific energy demand motive power** | | | | | | |
| --- | --- | --- | --- | --- | --- | --- |
|  | BAU | | POI | | ZEM | |
|  | **2019 [kWh/€]** | **eff.improve-ment [%/a]** | **2019 [kWh/€]** | **eff.improve-ment [%/a]** | **2019 [kWh/€]** | **eff.improve-ment [%/a]** |
| Chemical and petro-chemical | 0.50 | -0.14% | 0.50 | -0.25% | 0.50 | -0.32% |
| Non-ferrous metals | 0.40 | -0.14% | 0.40 | -0.25% | 0.40 | -0.32% |
| Non-metallic minerals | 0.45 | -0.14% | 0.45 | -0.25% | 0.45 | -0.38% |
| Transport equipment | 0.10 | -0.14% | 0.10 | -0.25% | 0.10 | -0.32% |
| Construction | 0.05 | -0.14% | 0.05 | -0.25% | 0.05 | -0.32% |
| Machinery | 0.07 | -0.14% | 0.07 | -0.25% | 0.07 | -0.32% |
| Mining and quarrying | 1.05 | -0.14% | 1.05 | -0.25% | 1.05 | -0.32% |
| Food and tobacco | 0.19 | -0.14% | 0.19 | -0.25% | 0.19 | -0.32% |
| Wood and wood products | 0.49 | -0.14% | 0.49 | -0.25% | 0.49 | -0.32% |
| Textile and leather | 0.23 | -0.14% | 0.23 | -0.25% | 0.23 | -0.38% |
| Industries not elsewhere specified | 0.15 | -0.14% | 0.15 | -0.25% | 0.15 | -0.38% |

Table A 4: Applied specific energy demand for lighting/IT applications.

| **Specific energy demand lighting/IT** | | | | | | |
| --- | --- | --- | --- | --- | --- | --- |
|  | BAU | | POI | | ZEM | |
|  | **2019 [kWh/€]** | **eff.improve-ment [%/a]** | **2019 [kWh/€]** | **eff.improve-ment [%/a]** | **2019 [kWh/€]** | **eff.improve-ment [%/a]** |
| Chemical and petro-chemical | 0.05 | -0.04% | 0.05 | -0.16% | 0.05 | -0.28% |
| Non-ferrous metals | 0.04 | -0.04% | 0.04 | -0.16% | 0.04 | -0.28% |
| Non-metallic minerals | 0.03 | -0.04% | 0.03 | -0.16% | 0.03 | -0.28% |
| Transport equipment | 0.03 | -0.04% | 0.03 | -0.16% | 0.03 | -0.22% |
| Construction | 0.01 | -0.04% | 0.01 | -0.16% | 0.01 | -0.22% |
| Machinery | 0.02 | -0.04% | 0.02 | -0.16% | 0.02 | -0.25% |
| Mining and quarrying | 0.06 | -0.04% | 0.06 | -0.16% | 0.06 | -0.25% |
| Food and tobacco | 0.02 | -0.04% | 0.02 | -0.16% | 0.02 | -0.25% |
| Wood and wood products | 0.05 | -0.04% | 0.05 | -0.16% | 0.05 | -0.25% |
| Textile and leather | 0.04 | -0.04% | 0.04 | -0.16% | 0.04 | -0.31% |
| Industries not elsewhere specified | 0.02 | 0.00 | 0.02 | -0.16% | 0.02 | -0.31% |

Table A 5: Applied specific energy demand for thermal applications.

| **Specific energy demand thermal** | | | | | | |
| --- | --- | --- | --- | --- | --- | --- |
|  | BAU | | POI | | ZEM | |
|  | **2019 [kWh/€]** | **eff.improve-ment [%/a]** | **2019 [kWh/€]** | **eff.improve-ment [%/a]** | **2019 [kWh/€]** | **eff.improve-ment [%/a]** |
| Chemical and petro-chemical | 1.25 | -0.32% | 1.24 | -0.49% | 1.24 | -0.61% |
| Non-ferrous metals | 0.76 | -0.32% | 0.76 | -0.49% | 0.76 | -0.61% |
| Non-metallic minerals | 2.22 | -0.32% | 2.21 | -0.49% | 2.21 | -0.65% |
| Transport equipment | 0.16 | -0.32% | 0.16 | -0.49% | 0.16 | -0.55% |
| Construction | 0.03 | -0.32% | 0.03 | -0.49% | 0.03 | -0.55% |
| Machinery | 0.11 | -0.32% | 0.11 | -0.49% | 0.11 | -0.58% |
| Mining and quarrying | 1.66 | -0.32% | 1.65 | -0.49% | 1.65 | -0.65% |
| Food and tobacco | 0.53 | -0.32% | 0.52 | -0.49% | 0.52 | -0.58% |
| Wood and wood products | 1.70 | -0.32% | 1.70 | -0.49% | 1.69 | -0.58% |
| Textile and leather | 0.29 | -0.32% | 0.29 | -0.49% | 0.29 | -0.58% |
| Industries not elsewhere specified | 0.13 | -0.32% | 0.13 | -0.49% | 0.13 | -0.58% |
